# Rise in Autism Spectrum Disorder Diagnoses: A Comparative Analysis of Pre-pandemic and Pandemic Periods

**DOI:** 10.7759/cureus.70167

**Published:** 2024-09-25

**Authors:** Rosemarie St. Victor, Srichand Mulakalapalli, Yohan Park, Emily Daly

**Affiliations:** 1 Pediatrics, Royale Pediatric Healthcare PC, New York, USA; 2 Pediatrics, Bayside Pediatrics PC, New York, USA; 3 Pediatrics, University of Limerick, Limerick, IRL

**Keywords:** autism spectrum disorder (asd), pandemic impact, pediatric diagnosis, pre-pandemic vs pandemic, retrospective data analysis

## Abstract

Introduction

This study analyzes the rise in autism spectrum disorder (ASD) diagnoses by comparing pre-pandemic (2017-2019) and pandemic period (2021-2023) data from two private pediatric practices with different populations in New York. The year 2020 was out of the analysis to focus on the impact after the shutdowns on ASD, and there were disruptions in clinic operations during that year. Clinic I primarily served an African-American ethnic population, while Clinic II primarily served an Asian population.

Methods

A retrospective analysis was conducted using de-identified patient numbers from the electronic medical records (EMR) of two private clinics. Only the numbers of new patients diagnosed each year were included from January 1, 2017, to December 31, 2019 (pre-pandemic), and January 1, 2021, to December 31, 2023 (pandemic). Sixteen ASD International Statistical Classification of Diseases and Related Health Problems 10th Revision (ICD-10) codes were included in this analysis. Descriptive and inferential statistics were used to find important patterns and determine statistical significance.

Results

The study included 537 patients, 182 from Clinic I and 355 from Clinic II. Clinic I demonstrated a significant increase in ASD diagnoses, from 63 (pre-pandemic) to 119 (pandemic) (χ2=17.23; p=0.000033). Clinic II demonstrated a significant increase in ASD diagnoses, from 149 (pre-pandemic) to 206 (pandemic) (χ2=9.15; p=0.00248).

Conclusion

The significant increase in ASD diagnoses in two private pediatric practices with different populations indicates a notable association with the shutdown periods and the pandemic. Factors such as disrupted routines, changes in access to healthcare services, and increased parental awareness may have contributed to this rise. Further longitudinal studies are needed to understand the long-term impacts of the pandemic on ASD diagnoses and care.

## Introduction

Autism spectrum disorder (ASD) is a developmental disorder with a prevalence of 1-2% across the United States. According to the DSM-5-TR, five criteria must be met to diagnose ASD. First, individuals must have ongoing social communication and interaction deficits across various settings. They must also exhibit restricted, repetitive behavior patterns, interests, or activities. Third, these symptoms need to start early in the developmental stage; however, they might not show up in full until social demands surpass their restricted abilities. Fourth, these symptoms must create considerable impairment in social, vocational, or other aspects of daily functioning. Finally, to diagnose someone with ASD, these symptoms cannot be better described by a global developmental delay or intellectual disability.

ASD can be further stratified into three levels based on the support needed due to the diagnosis. It may also be associated with difficulties with language and intellectual disability and other genetic, neurodevelopmental, or behavioral disorders. ASD is typically recognized between one and two years of age but can be identified earlier or later depending on the severity of the symptoms [1].

The COVID-19 pandemic and the ensuing lockdowns negatively affected people's mental health, and studies show that mental health was impacted not only during the pandemic lockdowns but also after the lockdowns [2]. In particular, there was an increase in anxiety and depression diagnoses after the pandemic, corresponding with a decrease in overall mental health [3]. One study indicated an increased prevalence of eating disorders, schizophrenia, mood disorders, and somatoform disorders following the pandemic [3]. Regarding ASD, the Centers for Disease Control and Prevention (CDC) stated that there is an increase in ASD prevalence after the COVID-19 pandemic compared to before [4]. The CDC also states that disruptions were made to the diagnosis of ASD during the pandemic, and children with ASD were not getting diagnosed as early as they were previously [4]. Due to the effects the COVID-19 pandemic had on mental health and different psychiatric illnesses, we hypothesize that the incidence of ASD diagnoses in children has increased after the pandemic. To investigate this, a de-identified retrospective analysis was conducted at two different pediatric private practices in New York, aiming for more variation in patient ethnicity and to validate the findings. Only the numbers of new patients from three years pre-COVID, 2017, 2018, and 2019, and three years post-COVID, 2021, 2022, and 2023, were included. The year 2020 was omitted due to the shutdown periods and potential disruptions in ASD diagnoses during that time. This research aimed to test the hypothesis that ASD diagnoses have increased following the COVID-19 pandemic.

This article was presented as a poster at the 2024 Michigan American Academy of Pediatrics Annual Conference on September 6, 2024.

## Materials and methods

Study design

A retrospective analysis was conducted using de-identified patient numbers extracted from the electronic medical records (EMR) of two private pediatric practices. Clinic I, Royale Pediatric Healthcare, New York, primarily serves an African-American population. Clinic II, Bayside Pediatrics, New York, primarily serves an Asian population. Only the numbers of new patients diagnosed with ASD each year during two distinct periods, pre-pandemic (January 1, 2017, to December 31, 2019) and pandemic (January 1, 2021, to December 31, 2023), were included in the analysis.

Inclusion criteria

Patients were included in the study if they were newly diagnosed with ASD during the pre-pandemic period from January 1, 2017, to December 31, 2019, and the pandemic period from January 1, 2021, to December 31, 2023. In addition, only patients whose diagnoses fell under one of the 16 ASD International Statistical Classification of Diseases and Related Health Problems 10th Revision (ICD-10) codes were included in the analysis. The 16 ASD ICD-10 codes were as follows: F84.8, F84.9, F80.9, F84.0, F80.0, F80.1, F80.2, F80.4, F80.81, F80.82, F84.5, R62.0, R62.50, R62.51, R62.52, and F80.89. Definitions of these ICD-10 codes are provided in the Appendices for further reference.

Exclusion criteria

Patients with a prior diagnosis of ASD before the study periods were excluded to ensure that only new diagnoses were analyzed. Patients whose diagnoses did not fall under one of the 16 ASD ICD-10 codes were excluded from the analysis.

Data collection

Only de-identified patient numbers were used, ensuring that no identifiable patient information was collected or analyzed. The study focused on the year-wise distribution of new ASD diagnoses across both clinics. The ethnic distribution percentages of the diagnosed patients were also analyzed to compare the differences between the two practices. However, no significant changes in ethnicity percentages were observed between the pre-pandemic and pandemic periods; thus, the total ethnic population from both practices was included in the analysis.

ASD screening and diagnosis

ASD diagnoses were made by pediatricians in both clinics following a multi-step process. First, pediatricians conducted a thorough developmental history of each child to identify any early signs or symptoms of ASD. Following the developmental history, the Modified Checklist for Autism in Toddlers [5], Revised (M-CHAT-R) was used as a screening tool to assess the risk of ASD in children aged 16-30 months. The M-CHAT-R is a validated parent-reported questionnaire that evaluates early signs of ASD through a 20-question checklist focused on areas such as social interaction, communication, and behaviors. Based on the responses, children were classified as low, medium, or high risk for ASD. This included a comprehensive examination by a pediatrician to confirm the diagnosis of ASD, with the diagnostic process based on the DSM-5 criteria. Once the diagnosis was confirmed, patients were assigned the appropriate ASD-related ICD-10 code(s) that corresponded to their specific condition.

Statistical analysis

All statistical analyses and visualizations were performed using the Python software, with the SciPy and Matplotlib libraries primarily utilized. Descriptive statistics were applied to summarize the number of new ASD diagnoses per year, as well as the ethnic distribution of diagnosed patients across both clinics. Additionally, inferential statistics were used to evaluate the significance of observed differences between the pre-pandemic and pandemic periods. The chi-squared test was conducted using SciPy to assess whether the variation in ASD diagnoses between these two periods was statistically significant. A p-value of less than 0.05 was considered statistically significant.

## Results

The study included 537 patients: 182 from Clinic I and 355 from Clinic II. The incidence of pre-pandemic and pandemic ASD diagnoses in the two clinics is shown in Table 1.

**Table 1:** Incidence of pre-pandemic and pandemic ASD diagnoses in the two clinics ASD: autism spectrum disorder

Year	Clinic I	Clinic II
2017	17	45
2018	22	51
2019	24	53
2021	41	71
2022	38	73
2023	40	62

Clinic I had 63 pre-pandemic ASD diagnoses and 119 pandemic ASD diagnoses. The chi-squared statistic for Clinic I was 17.23 with a p-value of 0.000033. For Clinic I, the mean number of pre-pandemic diagnoses per year was 21.0 (SD=3.6; range=17-24), and the mean number of pandemic diagnoses per year was 39.7 (SD=1.5; range=38-41) (Table 2). Clinic II had 149 pre-pandemic ASD diagnoses and 206 pandemic ASD diagnoses. The chi-squared statistic for Clinic II was 9.15 with a p-value of 0.00248. For Clinic II, the mean number of pre-pandemic diagnoses per year was 49.7 (SD=4.2; range=45-53), and the mean number of pandemic diagnoses per year was 68.7 (SD=5.9; range=62-73) (Table 2).

**Table 2:** Statistical analysis across two clinics

Clinic	Pre-pandemic diagnoses	Pandemic diagnoses	Chi-squared stats	P-value	Mean pre-pandemic Dx/year	Mean pandemic Dx/year
Clinic I	63	119	17.23	0.000033	21.0 (SD=3.6; range=1724)	39.7 (SD=1.5; range=3841)
Clinic II	149	206	9.15	0.00248	49.7 (SD=4.2; range=4553)	68.7 (SD=5.9; range=6273)

Among the 537 patients included in the study, Clinic I had a total of 182 patients, consisting of 131 males and 51 females. Clinic II had a total of 355 patients, consisting of 244 males and 111 females.

This study did not find any significant changes in ethnicity percentages between the pre-pandemic and pandemic periods. Therefore, the total ethnic population in both practices is presented: Clinic I primarily served an African-American population (92%), others (5%), and unknown (3%) ethnicities. Clinic II primarily served an Asian population (91%), others (3%), and unknown (6%) ethnicities (Table 3).

**Table 3:** Ethnic distribution of ASD diagnoses (combined pre-pandemic and pandemic data) ASD: autism spectrum disorder

Clinic	Ethnicity	Number (percentage)
Clinic I (total: 182 patients)	African-American	168 (92%)
	Other	9 (5%)
	Unknown	5 (3%)
Clinic II (total: 355 patients)	Asian	323 (91%)
	Other	11 (3%)
	Unknown	21 (6%)

These results are consistent with reports from the CDC [4] indicating an increase in ASD prevalence after the COVID-19 pandemic compared to before. This study observed an increase in the incidence of new ASD diagnoses during the pandemic period.

## Discussion

This study found a significant increase in ASD diagnoses in Clinic I and Clinic II during the pandemic period (2021-2023) compared to the pre-pandemic period (2017-2019). Clinic I showed an increase from 63 to 119 ASD diagnoses, while Clinic II showed an increase from 149 to 206 diagnoses.

Shutdown periods and restricted environments were some of the preventive measures followed by governments and the public during the COVID-19 pandemic. Numerous families worldwide experienced psychological effects as a result of these procedures [6,7]. In 2020, Amorim et al. conducted a cross-sectional study highlighting the potential psychological impact of the COVID-19 pandemic on children with neurodevelopmental disorders and their caregivers [7]. Increased anxiety and behavioral changes in children with ASD during the pandemic likely led to more parents seeking evaluations and diagnoses for their children [7]. In a 2021 research article, Bhat summarized that when the COVID-19 pandemic started, parents experienced significant interruptions in health services, which negatively impacted both their own mental health and the behaviors associated with ASD in their children [8]. These findings suggest that the disruption of routines and reduced access to services during the pandemic may have contributed to the increased incidence of ASD diagnoses observed in our study.

The pandemic lockdowns may have contributed to the identification of previously overlooked ASD cases. With families spending more time at home, parents had increased opportunities to observe developmental concerns in their children. This heightened awareness, combined with changes in healthcare access, likely led to more children being diagnosed during the pandemic than in prior years. Research suggested that telehealth services were effective during the pandemic as an assessment tool and in providing healthcare to ASD patients [9]. Therefore, telehealth services may also have played a role in the rise of ASD diagnoses during the pandemic. ASD prevalence has been reported to be higher among Asian ethnicity children [10], with the CDC also reporting that prevalence among Asian, Black, and Hispanic children was at least 30% higher in 2020 [11]. In our study, we observed significant results in both the African-American and Asian ethnic groups, suggesting that the pandemic may have influenced the rise in diagnoses across different populations.

Limitations

Due to its retrospective methodology and the fact that data was only collected from two particular clinics, this study has limitations. This could lead to selection bias and reduce the findings' generalizability to other areas or groups. The study does not account for all potential confounding factors, such as age, prenatal and postnatal factors, socioeconomic status, healthcare access, and changes in diagnostic criteria over time. Future research should address these limitations to offer a more comprehensive understanding of the factors influencing ASD diagnoses during the pandemic.

## Conclusions

The significant increase in ASD diagnoses in two private pediatric practices with different populations indicates a notable association with the shutdown periods and the pandemic. The lockdowns during the pandemic may have contributed to the identification of previously overlooked ASD cases, as parents had more opportunities to observe developmental concerns. Factors such as disrupted routines, changes in access to healthcare services, and increased parental awareness may have further contributed to this rise. Further longitudinal studies are needed to understand the long-term impacts of the pandemic on ASD diagnoses and care.

## Appendices

**Table 4:** ICD-10 codes used for ASD diagnosis and related developmental disorders ASD: autism spectrum disorder; ICD-10: International Statistical Classification of Diseases and Related Health Problems 10th Revision Reference: [12]

ICD-10 codes	
F84.8	Other pervasive developmental disorders
F84.9	Pervasive developmental disorder, unspecified
F80.9	Developmental disorder of speech and language, unspecified
F84.0	Autistic disorder
F80.0	Specific speech articulation disorder, phonological disorder
F80.1	Expressive language disorder
F80.2	Mixed receptive-expressive language disorder
F80.4	Speech and language developmental delay due to hearing loss
F80.81	Childhood-onset fluency disorder (stuttering)
F80.82	Social (pragmatic) communication disorder
F84.5	Asperger's syndrome
R62.0	Delayed milestone in childhood
R62.50	Unspecified lack of expected normal physiological development in childhood
R62.51	Failure to thrive (child)
R62.52	Short stature (child)
F80.89	Other developmental disorders of speech and language
